# An unusual case of tracheo-pleural fistula and cardiac metastases in oropharyngeal carcinoma: a case report and review of the literature

**DOI:** 10.1186/s41199-016-0018-5

**Published:** 2016-12-15

**Authors:** Daris Ferrari, Carla Codecà, Giulia Viale, Barbara Bocci, Francesca Broggio, Francesca Crepaldi, Martina Violati, Andrea Luciani, Dario Bauer, Laura Moneghini, Gaetano Bulfamante, Paolo Foa

**Affiliations:** 1 Medical Oncology, San Paolo Hospital, Milan, Italy; 2 Division of Pathology, San Paolo Hospital, University of Milan Medical School, Milan, Italy; 3 Department of Medicine, Surgery and Dentistry, Division of Pathology, San Paolo Hospital, University of Milan Medical School, Milan, Italy; 4 Department of Oncology, Medical Oncology, San Paolo Hospital, University of Milan Medical School, Milan, Italy; 5 San Paolo Hospital, via Di Rudinì 8, 20142 Milan, Italy

**Keywords:** Oropharyngeal carcinoma, Tracheo-pleural fistula, Cardiac metastases, Chemoradiotherapy, Risk factors

## Abstract

**Background:**

Oropharyngeal cancer is frequently associated with human papilloma virus, that also represents a strong prognostic factor. Local relaps and treatment-related complications are frequent, whereas distant metastases occur in about 25% of patients.

**Case presentation:**

A 49 years-old male presented with a loco-regionally advanced oropharyngeal squamous cell carcinoma and was treated with concomitant chemoradiation. A complete clinical and pathological response was achieved, but the occurrence of necrotising tracheo-esophagitis, with tracheo-mediastino-pleural fistula formation, further complicated the subsequent clinical course. The patient died suddenly. Autopsy revealed multiple myocardial and epicardial metastases from oropharyngeal squamous cell carcinoma.

**Conclusions:**

Even in case of a transient complete local response, the potential occurrence of severe complications and distant metastases, although infrequent, should be considered. Cardiac metastases are frequently underestimated, as they are often asymptomatic, but may lead to sudden death. Further efforts are needed to improve diagnosis and therapy in this setting.

## Background

Increasing oropharyngeal cancer incidence has been observed in numerous developed nations over the past few decades, though with marked difference between countries. It has been extensively demonstrated that alcohol intake and smoke are risk factors for head and neck cancer (HNC) development; recently, human papilloma virus (HPV), particularly HPV-16 subtype, was shown to be etiologically related to oropharyngeal tumors [1, 2].

In addition, a fair number of studies demonstrated the prognostic relevance of HPV, with a strikingly better prognosis and improved responsiveness to chemotherapy (CHT) and radiotherapy (RT) for HPV-positive oropharyngeal cancer [3].

Head and neck cancer tends to recur loco-regionally, and distant metastases are found in less than 30% of patients [4–7]. Common sites of distant metastases are lungs and liver, less frequently the central nervous system. Cardiac metastases from HNC are extremely rare [8, 9] and the few cases reported in literature are mainly found in patients affected by tongue cancer [10, 11]. Metastases to the heart and pericardium are much more common than primary cardiac tumors, originating in most cases from a carcinoma of the lung [12].The autoptic frequency of cardiac metastases varies from 0.7% to 3.5% in the general population, but may increase to 9.1% in patients with known malignancies [13–16]. The probability of cardiac involvement depends on the anatomy of the primary site, disease stage, intrinsic tumor and host biology. Tumors involving the heart are mainly represented by lung cancer (36-39% of cardiac metastases), breast cancer (10-12%), leukemias (10-21%), gastric, renal and pancreatic carcinoma, mesothelioma and melanoma [17–19]. Since cardiac metastases are usually clinically silent, they are frequently unrecognized and diagnosed only post-mortem.

## Case presentation

A 49-year-old male was admitted to our hospital in November 2012, with a two-month history of a sore throat and difficulties in swallowing. The oropharyngeal inspection revealed the presence of an ulcerated mass of the right tonsil extending to the base of the tongue, to the epiglottis and to the right pyriform sinus. In addition, bilateral neck lymphadenopaties were palpable. A biopsy was performed and an HPV- negative squamous cell oropharyngeal carcinoma was diagnosed. The patient was a heavy smoker (20 packs/year) but denied alcohol intake. Head and neck computed tomography (CT) scan and whole body fluorodeoxyglucose positron emission tomography/computed tomography (FDG PET/CT) scan confirmed the presence of tumor in the right tonsil area with bilateral lymph nodes involvement in levels I-III. The largest nodes measured 2-4 cm in maximum diameter. No distant metastases were found. The TNM clinical stage was cT3N2c. Following a multi-disciplinary discussion, the patient was judged suitable for concomitant chemoradiotherapy (CRT) and underwent protective tracheostomy; then, from April to May 2013, he was treated with a combination of intensity-modulated radiation therapy (IMRT) for a 70 Gy total dose, and weekly cisplatin for a total dose of 280 mg/m^2^.

The course of treatment was poorly tolerated, due to hematologic toxicity (grade 2 neutropenia) and grade 4 oropharyngeal mucositis, eliciting severe pain and requiring the placement of a percutaneous endoscopic gastrostomy (PEG) feeding tube. One month after the end of concomitant CRT, the patient’s clinical conditions slowly improved.

The CT scan performed at the end of treatment was negative, and a right tonsil biopsy did not reveal any residual cancer cell.

In July 2013, the patient was hospitalized because of the sudden onset of dysphagia, oral pain and extensive oral mycosis. Fiberoptic endoscopy revealed erosions and ulcerations of the tracheal, hypopharyngeal and esophageal mucosa, with fungal colonization. Biopsies confirmed the presence of Candida species hyphae and the absence of cancer cells, suggesting the diagnosis of a post-radiation tracheo-esophagitis with fungal colonization.

**Fig. 1:**
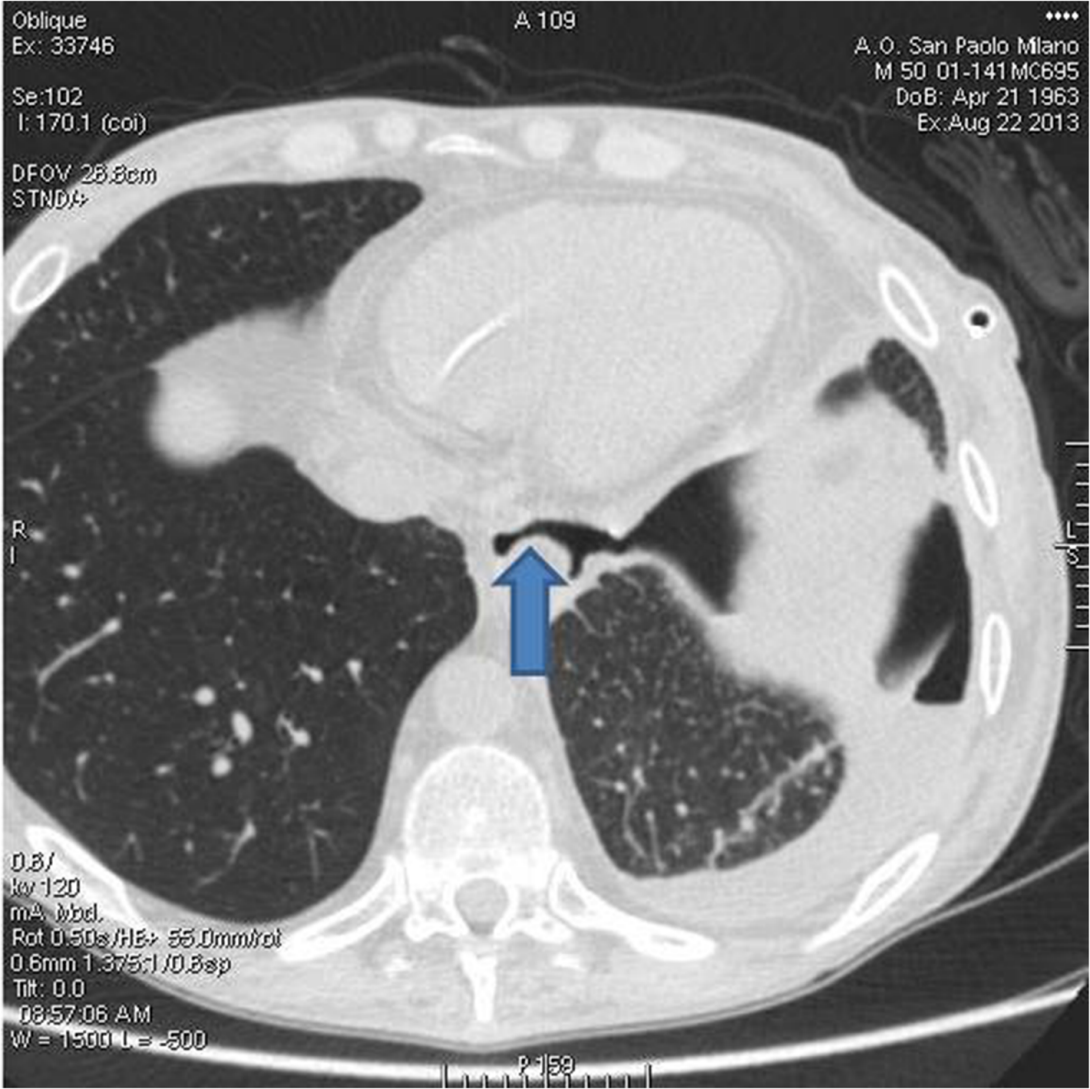
Contrast-enhanced CT-scan showing an air-filled communication between trachea and mediastinum

A few days later the patient, still in treatment with analgesic and antifungal drugs and fed through the PEG, reported a stabbing right hemithoracic pain. A plain chest X-ray showed the occurrence of a spontaneous pyo-pneumothorax that was treated by pleural purulent fluid drainage and broad-spectrum antibiotics. During the following days, as a persistent discharge of dark purulent fluid from the pleural drainage was noted, enteral nutrition reflux from the stomach to the esophagus, and then along the upper airways to the mediastinum and the pleural cavity, was suspected, possibly due to the formation of a tracheo-mediastino-pleural fistula. The diagnosis was confirmed by a CT scan (Fig. 1) and the fistula was treated with the endoscopic insertion of a silicone stent.

In October 2013, a total-body CT scan was performed; it showed no evidence of malignant disease and the resolution of the fistula. Two months later, the patient was urgently hospitalized for loss of consciousness secondary to hypotension. Anemia (hemoglobin 8 g/dL), probably caused by a minor oropharyngeal bleeding from the tracheostoma, was observed in blood tests. A thoracic CT scan, the electrocardiogram and a two-dimensional echocardiogram (ECHO) revealed normal findings with the exception of the presence of sinus tachycardia. The patient died suddenly two days after hospitalization.

**Fig. 2:**
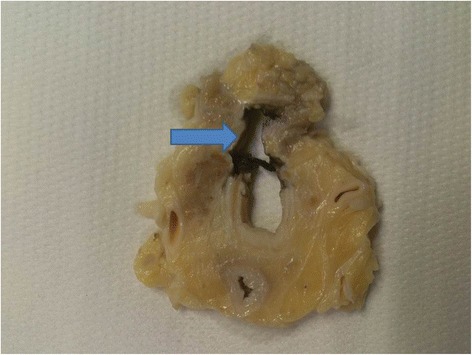
Transversal section of the trachea with evidence of wall disruption

**Fig. 3:**
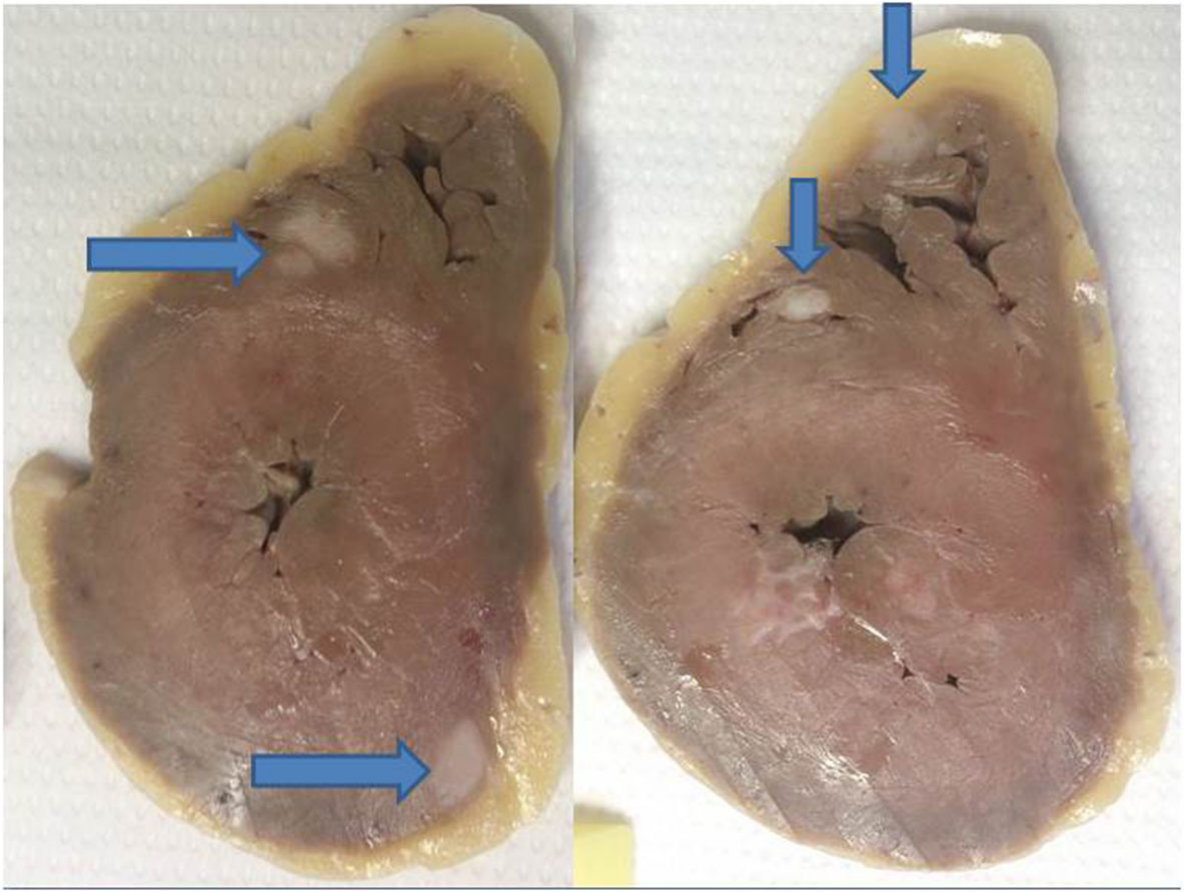
Sections of the hearth at ventricular level, with evidence of metastases at the interventricular septum and the walls of the right and the left ventricle

**Fig. 4:**
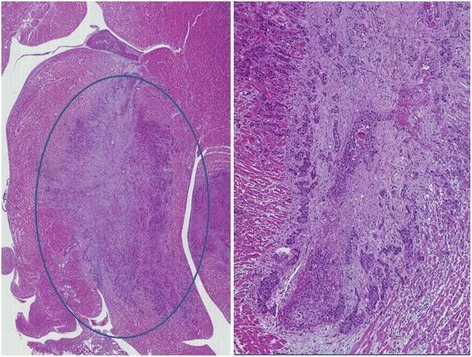
*Left*: low power picture of the wall of left ventricle with evidence of metastatic infiltration (Hematoxylin & Eosin, 4X). *Right*: the same field at 10X

The autopsy revealed sparse tumor cells in the oropharyngeal and tracheal mucosa, a minimal residual tracheo-esophageal fistula, and a large number of epicardial and myocardial metastases from oropharyngeal squamous cell carcinoma (Figs. 2, 3 and 4).

## Conclusions

Patients affected by oropharyngeal cancer have a slightly better prognosis compared to different sites HNC patients, but severe clinical complications, either due to the tumor itself or to loco-regional and systemic therapy, can seriously compromise survival and quality of life. The poor prognosis in our case might be influenced by the negativity of HPV status and by the presence of well- known risk factors such as heavy smoking, implying a more aggressive disease [3]. However such a series of events is unusual in oropharyngeal cancer, particularly when loco-regional control of the tumor has been achieved.

In our case a tracheo-mediastino-pleural fistula formation unexpectedly altered the course of the disease after concomitant CRT. Documented complications of HNC surgery are mainly pharyngocutaneous fistulae [20]. Tracheo-pleural fistulae are rare consequences of lung surgery; esophago-pleural fistulae have been reported as post-surgical complications in a case of peptic ulcer perforation, in variceal sclerotherapy, or secondary to infections, usually tuberculosis or mycosis [21–24].

To our knowledge, this is the first report of tracheo-mediastino-pleural fistula occurrence in a patient treated with concomitant CRT for HNC. In our case, the occurrence of the fistula was probably due to a combination of tumor spread, Candida mycosis, and inflammatory reaction sustained by cytokines production. A case of tracheo-pleural fistula has been reported as a consequence of chemoradiation and bevacizumab therapy in non-small cell lung cancer; it was successfully treated by the endoscopic insertion of a silicone stent covered by a metal sheath, as in our case [25].

Although the endoscopic treatment of a fistula is feasible and often successful, cardiac dissemination of HNC is almost invariably fatal, leading sometimes to sudden death. In our case the occurrence of cardiac metastases further complicated the patient’s clinical course and probably was the cause of his death.

Cardiac metastases are rarely diagnosed *ante mortem* as they are frequently asymptomatic, but they are found in up to 24% of autopsied patients, mainly affected by tongue cancer [26]. Head neck cancer has limited propensity to give rise to distant metastases, and only sporadic cases of cardiac involvement have been reported, particularly in advanced stage [27–30], although it has been occasionally observed also in the absence of local recurrence [31]. Metastatic spread may be due to dissemination in the bloodstream or in the lymphatic system, or to intracavitary diffusion through the inferior vena cava or pulmonary veins [18].

Cardiac metastases cause non-specific symptoms that depends on the size and location of the metastases, mimicking a myocardial infarction, or presenting with ventricular tachycardia, pericardial effusion and cardiogenic shock [27–29, 32, 33]. However, cardiac metastases do not generally lead to clinical findings at an early stage and may be diagnosed, if suspected, by ECHO and cardiac Magnetic Resonance (MR) or CT scan [34–36]. In our case the ECHO was negative for metastases shortly before the onset of the terminal symptoms leading to sudden death. An FDG PET/CT scan was not repeated, as a consequence of the absence of metastases on CT scan. It would be of interest to evaluate the presence of increased FDG activity in the form of cardiac deposits, even in case of negative CT, but the rapid and fatal course in our case prevented further clinical evaluations. Cardiac MR is a validated tool for tissue characterization of cardiac masses, but prospective trials with the aim of studying the prognostic implications of cardiac metastases among patients with systemic disease are lacking [37].

The treatment of cardiac metastases is generally ineffective and cardiac dissemination is almost invariably fatal, as previously reported [30, 33, 38].

The rare complications reported in this case highlight the need to carefully monitor the clinical evolution and the onset of new symptoms in all patients affected by HNC.

## Abbreviations

CRT: Chemo-radiotherapy
CT: Computed tomography
FDG: PET/CT Fluorodeoxyglucose positron emission tomography/computed tomography
HNC: Head and neck cancer
HPV: Human papilloma virus
IMRT: Intensity-modulated radiation therapy
MR: Magnetic resonance
PEG: Percutaneous endoscopic gastrostomy
